# Genetic control of root architectural plasticity in maize

**DOI:** 10.1093/jxb/eraa084

**Published:** 2020-02-21

**Authors:** Hannah M Schneider, Stephanie P Klein, Meredith T Hanlon, Eric A Nord, Shawn Kaeppler, Kathleen M Brown, Andrew Warry, Rahul Bhosale, Jonathan P Lynch

**Affiliations:** 1 Department of Plant Science, The Pennsylvania State University, University Park, PA, USA; 2 Department of Agronomy, University of Wisconsin, Madison, WI, USA; 3 Advanced Data Analysis Centre, University of Nottingham, Nottingham, UK; 4 Plant and Crop Sciences, School of Biosciences, University of Nottingham, Sutton Bonington, UK; 5 Lancaster University, UK

**Keywords:** Architecture, association mapping, maize, plasticity, root, water deficit stress

## Abstract

Root phenotypes regulate soil resource acquisition; however, their genetic control and phenotypic plasticity are poorly understood. We hypothesized that the responses of root architectural phenes to water deficit (stress plasticity) and different environments (environmental plasticity) are under genetic control and that these loci are distinct. Root architectural phenes were phenotyped in the field using a large maize association panel with and without water deficit stress for three seasons in Arizona and without water deficit stress for four seasons in South Africa. All root phenes were plastic and varied in their plastic response. We identified candidate genes associated with stress and environmental plasticity and candidate genes associated with phenes in well-watered conditions in South Africa and in well-watered and water-stress conditions in Arizona. Few candidate genes for plasticity overlapped with those for phenes expressed under each condition. Our results suggest that phenotypic plasticity is highly quantitative, and plasticity loci are distinct from loci that control phene expression in stress and non-stress, which poses a challenge for breeding programs. To make these loci more accessible to the wider research community, we developed a public online resource that will allow for further experimental validation towards understanding the genetic control underlying phenotypic plasticity.

## Teaser

Root architectural phenes have heritable and plastic responses, and genetic loci associated with stress and environmental plasticity are distinct from loci controlling phenotypic expression in water-stress and well-watered conditions.

## Introduction

Crop varieties are generally developed for specific environmental and management scenarios. However, increasingly unpredictable growth environments due to climate change, decreasing freshwater availability, and rising costs of fuel and nitrogen fertilizer require the development of crop varieties that are resistant to abiotic stress and for increased production in marginal soils (Tebaldi and Lobell, 2008; Brisson *et al.*, 2010; Woods *et al.*, 2010; Sandhu *et al.*, 2016). The occurrence of water deficit stress is likely to become increasingly frequent and unpredictable as a result of global climate change. Phenotypic plasticity is the ability of a plant to alter its phenotype in response to the environment and encompasses components of genotype by environment interaction (G×E). Plasticity may be morphological, anatomical, and developmental, involve changes in resource allocation (Sultan, 2000), and is under genetic control (e.g. Sandhu *et al.*, 2016). Breeding programs have prioritized uniformity and yield stability in specific environments, and phenotypic plasticity has often been considered a challenge in this context (Basford and Cooper, 1998; Cooper *et al.*, 1999). It has been proposed that crops that can adapt their growth in response to environmental signals may be a breeding target for increasing agricultural productivity (e.g. Nicotra *et al.*, 2010; Topp, 2016), although the fitness impacts of phenotypic plasticity are poorly understood, and it has been proposed that plasticity may be maladaptive in some cases (Lynch, 2013, 2018).

Root architectural phenes (‘phene’ is to ‘phenotype’ as ‘gene’ is to ‘genotype’) (Lynch, 2011; Pieruschka and Poorter, 2012; York *et al.*, 2013) have important roles in soil resource capture, particularly in environments with suboptimal water and nutrient availability. Root architectural phenes determine the temporal and spatial distribution of roots in specific soil domains and their ability to obtain mobile and immobile resources (Lynch, 1995, 2013, 2019; Hirel *et al.*, 2007; Lynch and Brown, 2012). For example, root growth angle influences root distribution and depth, and therefore plant performance in nutrient and water-deficit stress conditions (Bonser *et al.*, 1996; Uga *et al.*, 2011; Trachsel *et al.*, 2013; York *et al.*, 2013; Dathe *et al.*, 2016). Root phene states that enable exploration of deep soil domains enhance the capture of mobile soil resources, including water and nitrate (Lynch and Wojciechowski, 2015). Steep growth angles enable deeper rooting and the capture of mobile nutrients, such as nitrogen, in deep soil domains (Trachsel *et al.*, 2013; Dathe *et al.*, 2016), while shallow growth angles are more beneficial for the capture of immobile resources in the topsoil, such as phosphorus (Bonser *et al.*, 1996; Lynch and Brown, 2001; Ho *et al.*, 2005; Zhu *et al.*, 2005*a*). Lateral root branching length and density have a significant effect on plant performance in water-stressed and low nitrogen environments, where longer, more dispersed lateral root branches are beneficial for the capture of mobile resources due to reduced inter- and intraplant competition for soil resources (Zhan and Lynch, 2015; Zhan *et al.*, 2015), while greater density of lateral branching improves topsoil foraging and phosphorus capture (Postma *et al.*, 2014; Jia *et al.*, 2018). Reduced crown root number improves plant growth in low nitrogen (Saengwilai *et al.*, 2014) and drought (Gao and Lynch, 2016) by reducing inter- and intraplant competition for internal and external resources, thereby increasing root depth and acquisition of deep soil resources. A reduced number of crown roots in modern maize lines increased plant growth in high nitrogen environments and was associated with increased nitrogen use efficiency compared with commercially successful lines a century ago (York *et al.*, 2015). Greater crown root number improves plant growth in low phosphorus soil by reducing axial root elongation and improving topsoil foraging (Sun *et al.*, 2018).

Root plasticity also varies spatially in response to soil conditions. In some genotypes and species, lateral root branches proliferate in response to localized patches of nitrogen and phosphorus availability (Drew, 1975; Zhu and Lynch, 2004) which has been proposed as a beneficial strategy for enhanced nitrogen acquisition (Mi *et al.*, 2010). However, this response may be maladaptive if mobile resources, such as nitrogen or water, move faster through the soil profile than roots can proliferate. This is especially detrimental when proliferation in one soil domain diverts resources from other soil domains that will have greater resource availability later in the season, for example deeper soil domains in leaching precipitation regimes (Lynch, 2013, 2018). The plasticity of lateral root branching in response to local nutrient patches may be beneficial for nutrient resource capture in environments where the nutrient source is sustained or in conditions of interspecific competition (Robinson *et al.*, 1999).

Phenotypic changes as a result of plasticity may be of short or long duration. For example, expression of nitrate transporters fluctuates rapidly in response to environmental signals including light and nitrogen availability (Feng *et al.*, 2011). In contrast, initial root growth angles are established after root emergence, and possible changes to morphology are limited in mature tissue. Plasticity of root phenes that are established early in development, such as root growth angle, may be beneficial in conditions of sustained edaphic stress, such as low phosphorus availability, but may be maladaptive for stresses that fluctuate on shorter time scales, including drought, by generating sustained responses to ephemeral conditions (Lynch, 2013).

Although root architectural phenes can improve the capture of soil resources in specific environments, for example sustained nitrogen or phosphorus stress (Saengwilai *et al.*, 2014; Dathe *et al.*, 2016), some phene states can be functionally maladaptive in fluctuating environments (Ho *et al.*, 2005; Poot and Lambers, 2008). In the field, the plant may be exposed to multiple, simultaneous, or successive stresses. For example, root phene states that improve topsoil foraging (e.g. shallow growth angle) are advantageous for phosphorus acquisition, but may be unfavorable for the capture of deep soil resources such as water (Ho *et al.*, 2005). Trade-offs also exist for phene states for nitrogen and phosphorus acquisition. For example, in common bean (*Phaseolus vulgaris*), shallow growth angle and a greater number of basal root whorls and hypocotyl-borne roots increase total root length in the topsoil, resulting in greater phosphorus uptake (Rangarajan *et al.*, 2018). However, as the number of axial roots and/or basal root whorls increase, the resulting carbon limitation leads to a reduced root depth and therefore trade-offs for nitrogen acquisition (Rangarajan *et al.*, 2018). No single root phenotype is optimal across a range of environments (Tardieu, 2012; Dathe *et al.*, 2016; Rangarajan *et al.*, 2018). Understanding phenotypic plasticity and its genetic control will be useful in developing strategies to optimize soil resource capture under multiple, dynamic stresses.

Phenotypic plasticity may improve plant performance in variable environments; however, in high-input environments with intensive fertilization and greater nitrogen and phosphorus availability, root plasticity may be counterproductive. Crops and crop ancestors evolved in ecosystems with one or more edaphic stresses influencing growth and root function. Therefore, the ancestral strategies for soil resource capture may not be useful in high-input environments in which constraints to root function are mitigated (Lynch, 2018). Root phenotypes that explore deep soil domains, whether plastic or not, may be beneficial in most environments for the capture of water and nitrogen. In the majority of agricultural systems, deeper root phenotypes enhance water and nitrogen capture, despite the fact that water and nitrogen availability are sometimes greater in surface soils of high-input systems (Manschadi *et al.*, 2006; Gowda *et al.*, 2011; Henry *et al.*, 2011).

Plasticity in root architecture may be advantageous for drought tolerance (e.g. Kano-Nakata *et al.*, 2013; Prince *et al.*, 2017). In drought conditions, plasticity in lateral root length and density (Kano *et al.*, 2011; Kano-Nakata *et al.*, 2013), root length density, and total root length (Kano-Nakata *et al.*, 2011; Tran *et al.*, 2014) correlated with greater shoot biomass, water uptake, and photosynthesis in rice. Plasticity in response to water deficit has also been observed for the number of nodal roots in rice (Suralta *et al.*, 2010) and maize (Gao and Lynch, 2016), lateral branching density and length in maize (Zhan *et al.*, 2015), and deep rooting in wheat (Ehdaie *et al.*, 2012; Wasson *et al.*, 2012), millet (Rostamza *et al.*, 2013), rice (Hazman and Brown, 2018), and maize (Nakamoto, 1993). Plasticity in the positioning of lateral branches, root hairs, and aerenchyma towards available water has also been documented as a phenomenon called hydropatterning (Bao *et al.*, 2014; Orosa-Puente *et al.*, 2018). In addition, high yield stability correlated with high root plasticity in drought and low phosphorus environments in rice (Sandhu *et al.*, 2016). Maize genotypes that increased root hair length in response to low phosphorus availability had better performance under low P than lines with constitutively long root hairs (Zhu *et al.*, 2010). A few previous studies have demonstrated that genes associated with phene expression may be distinct from those associated with plasticity for that expression. Genetic regions controlling plasticity have been identified for root hair length (Zhu *et al.*, 2005*b*) and lateral root branching and length (Zhu *et al.*, 2005*c*) in maize under low phosphorus availability, and for lateral root growth (Niones *et al.*, 2015), root anatomy (Kadam *et al.*, 2017), and root length density and root dry weight (Sandhu *et al.*, 2016) in rice in response to drought. The identification of genetic regions controlling plasticity could provide useful breeding targets for crop improvement and may aid in understanding the benefits and trade-offs of root plasticity (Collins *et al.*, 2008).

The objectives of this research were to test the hypotheses that (i) the responses of root architectural phenes to water deficit (stress plasticity) and different environmental conditions (environmental plasticity) are under genetic control; and (ii) genetic loci associated with plasticity are distinct from loci controlling phenotypic expression in water-stress and well-watered conditions. Here we identify and characterize phenotypic plasticity in root architectural phenes in mature, field-grown maize and identify distinct genetic regions controlling these phenes in well-watered and water-stress conditions as well as genetic regions controlling the plastic stress and environmental response of these phenes.

## Materials and methods

### Field conditions, experimental design, and plant materials

Root architecture phenotypes were measured on the Wisconsin Diversity Panel (Hansey *et al.*, 2011). The Wisconsin Diversity Panel is a large association panel composed of inbred maize lines that reach grain physiological maturity in the upper Midwest region of the USA and display uniformity and vigor. Experiments were conducted at the Apache Root Biology Center (ARBC) in Willcox, Arizona (32°153' 9.252''N, 109° 49' 56.928'' W) in well-watered (ARBC-WW) and water-stress (ARBC-WS) conditions (Supplementary Table S1 at *JXB* online, 383 genotypes planted) and at the Ukulima Root Biology Center (URBC) in Alma, Limpopo, South Africa (24°33'0012''S, 28°07'2584''E) under non-stress conditions (Supplementary Table S2, 641 genotypes planted). The Arizona experiments were conducted on a Grabe loam (coarse-loamy, mixed, thermic Typic Torrifluvent) from May to September 2014, 2015, and 2016. Genotypes were grown in two replications per treatment in a randomized complete block design each year. The experiments in South Africa were conducted on a Clovelly loamy sand (Typic Ustipsamment) from January to April in 2010, 2011, and 2012, and from November to February in 2013. Genotypes were grown in four replications in a randomized complete block design. For all experiments, each line was planted in a single row plot consisting of 20 plants per plot. Row width was 75 cm and distance between plants within a row was 23 cm. Soil nutrient levels were adjusted based on soil tests at the beginning of the season to meet the requirements for maize production. Pest control was carried out as needed. In South Africa, trials were irrigated using a center pivot system. In Arizona, trials were irrigated using drip irrigation in 2014 and a center pivot system in 2015 and 2016, and drought and well-watered treatments were grown in separate blocks. Water stress was confirmed by an ~20% vegetative biomass growth reduction and 40% yield reduction in water-stressed compared with well-watered conditions. Drought was induced ~4 weeks after planting. Drought was monitored throughout the growth season by PR2 multi-depth soil moisture probes (Dynamax, Houston, TX, USA).

### Phenotypic analysis

Root architecture was phenotyped in all experiments. Evaluations of maize root crowns for architecture were performed based on the shovelomics method followed by manual phenotyping (Trachsel *et al.*, 2011) in 2010–2012 and image analysis with Digital Imaging of Root Traits (DIRT) in 2013–2017 (Bucksch *et al.*, 2014; Das *et al.*, 2015). At anthesis, three representative plants were excavated from each plot for architectural analysis from 2010 to 2014, and one representative plant in 2015–2016. In brief, root crowns were excavated in a soil monolith using a standard shovel. Root crowns were soaked in water for 15 min to remove soil. The root crowns were then washed with low-pressure water to remove remaining soil. Four root architectural phenes were collected (Table 1) by imaging or manually phenotyping cleaned root crowns. Average lateral root length (LL), lateral branching frequency on the excised root (BF), and root angle (ANGLE) were measured in all experiments. Distance to the first lateral branch (DISTLAT) was only collected in 2013–2016. Excised root traits were measured on a representative third whorl crown root at the South Africa field site and on a representative fourth whorl crown root at the Arizona field site. Plant height at anthesis was measured in three plants per plot at anthesis in South Africa. Shoot dry biomass was collected for one plant per plot at anthesis in Arizona. Yield was collected at physiological maturity, and cobs from three plants per plot were bulked and weighed.

**Table 1.** Description of architectural phenes measured at anthesis

Trait	Description	Units
LL	Average lateral root length	mm
DISTLAT	Distance to the first lateral root from the root apex on the excised root	mm
BF	Lateral branching frequency on the excised root	Branches mm^–1^
ANGLE	Angle of roots relative to the soil line	°

‘Excised root’ is a representative third whorl crown root in Arizona and a representative second whorl crown root in South Africa.

### Data analysis

Plasticity in response to water deficit was calculated as a relative value compared with control growing conditions for each phene under no stress:

$$\begin{array}{l} Stress\;plasticity=\frac{\left(WS-WW\right)}{WW} \end{array}$$

where water stress (WS) is the mean value of the phene in water-stress conditions for each replication and well-watered (WW) is the mean value of the phene in well-watered conditions for each replication.

In the case of environmental plasticity, plasticity was calculated as a relative phenotypic value of South Africa growing conditions compared with the Arizona growing conditions for each phene:

$$\begin{array}{l} Environmental\;plasticity=\frac{\left(SA-AZ\right)}{AZ} \end{array}$$

where SA is the mean value of the phene in the South Africa environment and AZ is the mean value of the phene in the Arizona environment.

Broad-sense heritability on an entry mean basis was calculated for each architectural phene according to Fehr (1993).

Spearman and Pearson correlations between replications and years suggested data could be combined by environment and treatment, therefore mean phenotypic values across all years were calculated and used for subsequent analysis. For phenotypes in water-stress and well-watered environments in Arizona, an average of two replications over 3 years within each treatment were combined. Plastic responses to water deficit were calculated by replication. For phenotypes in well-watered environments in South Africa, the averages of four replications over 4 years were combined. Plastic responses to the environment were calculated by year. Residuals were transformed according to boxcox analysis.

Architectural phenotypes in well-watered and water-stress conditions and their corresponding plasticity values were used in a Multiple Loci Linear Mixed Model for genome-wide association study (GWAS) analysis (Zhang *et al.*, 2010) implemented in the FarmCPU R package (X. Liu *et al.*, 2016). The model used 591 688 single nucleotide polymorphism (SNP) markers (Mazaheri *et al.*, 2019). Allelic effects are estimated relative to the minor allele.

Significant SNPs were identified based on a genome-wide corrected Bonferroni threshold of –log(*P*)=7.07 based on the number of SNP markers used in the model. QQ-plots for each phene suggested a good model fit (Supplementary Fig. S1).

R Software (version 3.2.4) (R Core Team, 2018), Bioconductor (Bates *et al.*, 2002), MapMan (Usadel *et al.*, 2009), and MaizeGDB (Lawrence, 2005) were used to annotate genes and compare significant SNPs across treatments. Significant differences in MapMan ontologies between treatments were determined using a Student’s *t*-test. Candidate genes identified through significant GWAS hits were detected based on the physical position of genes in the version 4 B73 (AGPv4) reference sequence assembly (Jiao *et al.*, 2017). To understand the functional relevance of associated candidate genes with root architectural traits, we examined the functional annotation and root expression of maize gene models and their respective orthologs in the genetically well-studied model plant *Arabidopsis thaliana*. In the WiDiv population, most LD mapping interval sizes are <2 kb (Hirsch *et al.*, 2014); therefore, we only considered genes which had significant SNPs to be candidate genes and did not consider neighboring genes. We used Plaza 4.0 monocots (Van Bel *et al.*, 2018) to determine one-to-one or one-to-many orthologs in *A. thaliana*, and TAIR (Rhee *et al.*, 2003) and the Arabidopsis eFP Browser (Winter *et al.*, 2007) to obtain ortholog function and its root tissue and root developmental expression patterns (Brady *et al.*, 2007).

## Results

**Fig. 1.**
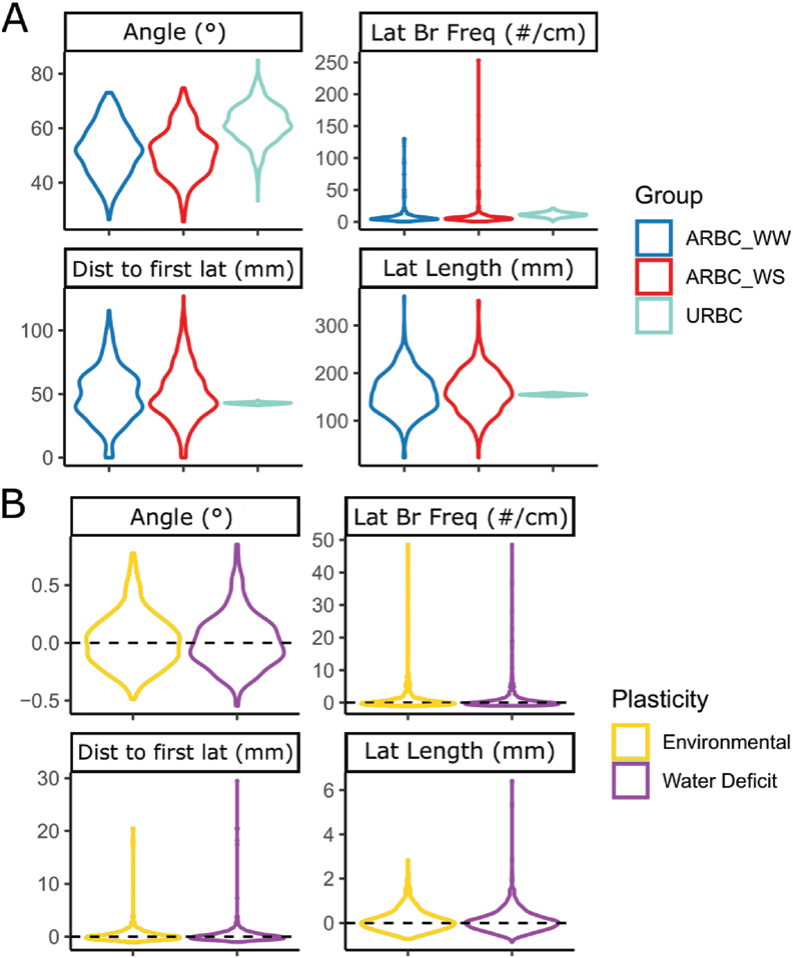
Distributions of genotypic means for each phene in (A) well-watered and water-stress conditions. (B) Distribution of the root phene stress and environmental plasticity. The *y*-axis represents the phene value in (A) and the relative difference in phene value between well-watered and water-stressed (stress plasticity) or relative difference between each environment (environmental plasticity) for each phene (B).

**Fig. 2.**
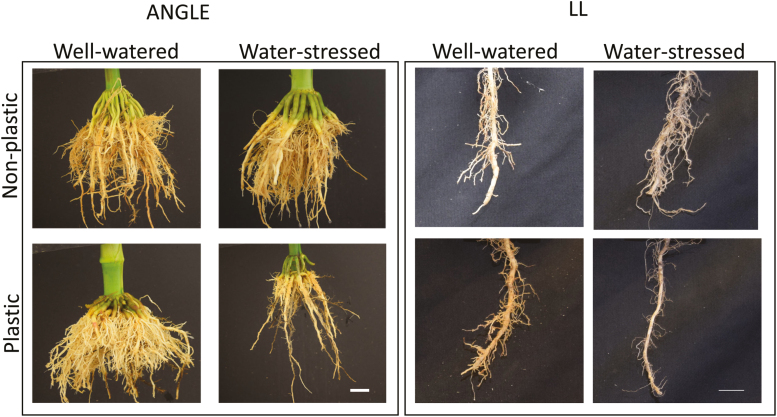
Genotypes vary in their plastic response to drought. Images of root crowns of a non-plastic genotype and a plastic genotype from well-watered and water-stress treatments. Architectural plasticity is shown for root angle and lateral branching length. Scale bar represents 1 cm (lateral branching length) and 2 cm (angle).

Plastic responses to drought and environment varied by phene (Fig. 1) and genotype (Fig. 2) for all four root phenes measured [angle (ANGLE), lateral branching frequency (BF), average lateral root length (LL), and distance to the first lateral branch (DISTLAT)] (Table 1). A wide range of natural variation was observed for architectural phenes, particularly in the Arizona environment (Supplementary Table S3). Water regime and environment had a significant effect on most root phenes (Supplementary Table S4) and water deficit on average reduced vegetative biomass by 21% and yield by 40% (Supplementary Table S3). LL was 4% greater in the Arizona environment compared with the South Africa environment, but no change in the phenotypic expression was observed between water-stress and well-watered conditions in the Arizona environment. In Arizona, phenotypic expression of nodal root angle (ANGLE), BF, and DISTLAT did not change between well-watered and water-stress conditions. BF and ANGLE were reduced by 46% and 38%, respectively in the Arizona environment compared with the South Africa environment. DISTLAT was 65% greater in the Arizona environment compared with the South Africa environment (Fig. 1). Expression of environmental and stress plasticity was not driven by a few genotypes, and most genotypes expressed plasticity to some degree. However, distinct genotypes expressed plasticity to different degrees for different phenes. Allometric relationships between root phenes and yield or vegetative biomass were not significant (Supplementary Table S5).

In both environments, LL and DISTLAT in well-watered and water-stress conditions were more heritable than their plastic responses (Table 2). The total phenotypic variation explained by the significant SNPs accounted for 36% of the variation of individual phenes. Most root architectural phenes were under highly quantitative genetic control, as demonstrated by the large number of significant SNPs identified with relatively small effect sizes (Table 2; Supplementary Tables S6–S10). Heritability for root architectural phenes was relatively low to moderate, ranging from 0.13 to 0.68 (Table 2).

**Table 2.** Heritability of root architectural phenes in the Wisconsin Diversity panel

			LL	DISTLAT	BF	ANGLE
**Arizona**	**Well-watered**	**Heritability**	0.19	0.21	0.13	0.16
		**% Variation explained by SNPs**	25.09	19.31	163.84	23.18
	**Water stress**	**Heritability**	0.15	0.17	0.16	0.22
		**% Variation explained by SNPs**	23.83	4.33	53.55	6.31
	**Stress plasticity**	**Heritability**	0.14	0.13	0.17	0.18
**South Africa**	**Well-watered**	**Heritability**	0.43	0.68	0.42	0.64
		**% Variation explained by SNPs**	24.77	6.56	72.34	4.69
	**Environmental plasticity**	**Heritability**	0.27	0.32	0.45	0.37

Broad-sense heritability on an entry mean basis and percentage variation explained by SNPs is the absolute sum of allelic effects of all significant SNPs for each phene. Explanations of abbreviations are given in Table 1.

**Fig. 3.**
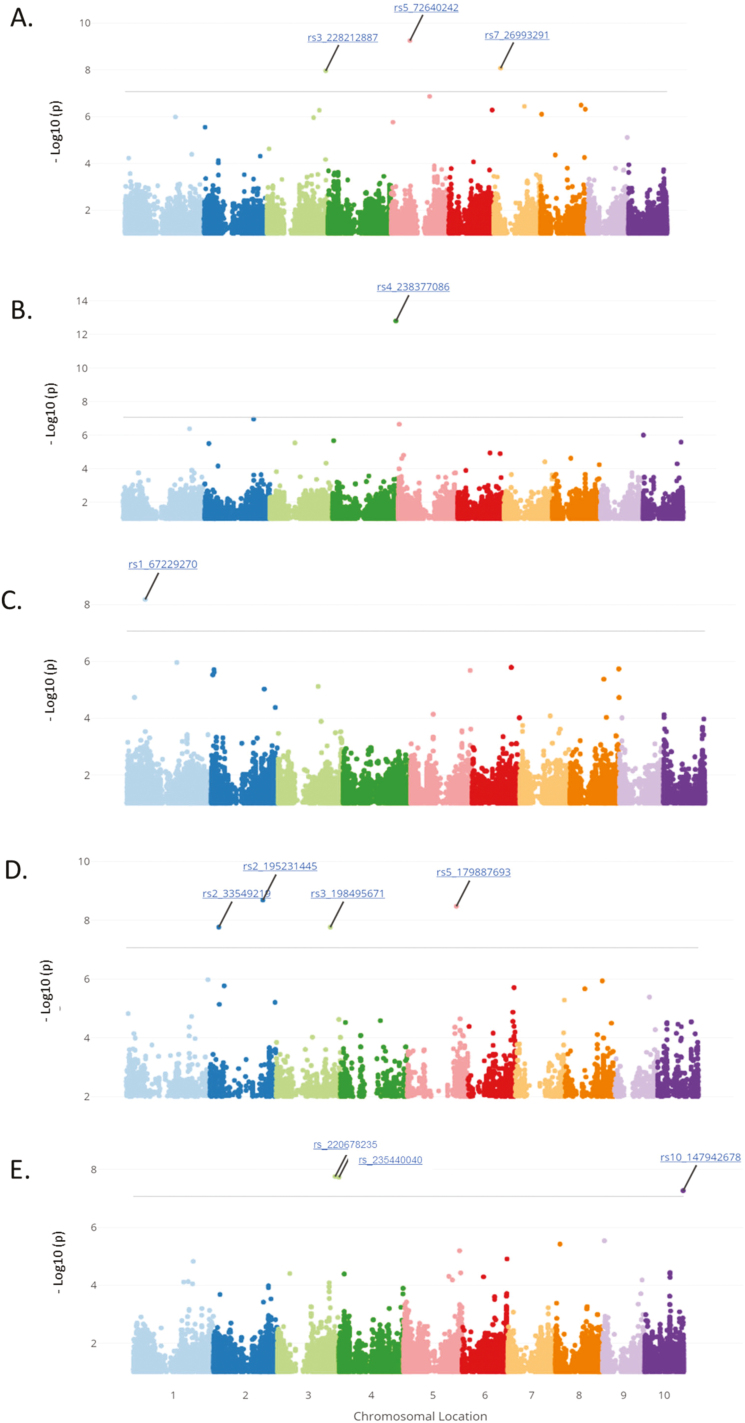
GWAS results for root angle (ANGLE) for plants grown in (A) well-watered conditions, (B) water-stressed conditions, (C) water-stress plasticity in Arizona, (D) well-watered conditions in South Africa, and (E) environmental plasticity. See Supplementary Figs S2–S4 for plots of other architectural phenes.

**Fig. 4.**
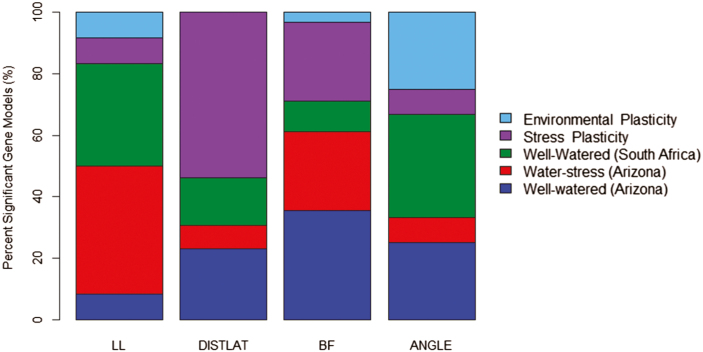
Relative proportion of unique gene models associated with well-watered, water-stress, stress plasticity, and environmental plasticity.

**Fig. 5.**
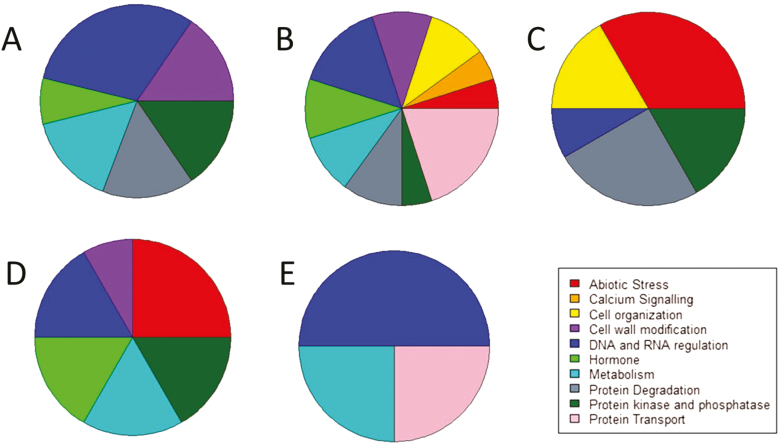
Mapman ontogenic categories for annotated gene models associated with significant SNPs in (A) water stress in Arizona (57% annotated), (B) well-watered in Arizona (48% annotated), (C) well-watered in South Africa (70% annotated), (D) stress plasticity in Arizona (29% annotated), and (E) environmental plasticity (80% annotated).

GWAS identified 69 significant SNPs associated with root architecture in well-watered and water-stressed plants and stress and environmental plastic responses, using a Bonferroni-corrected genome-wide threshold value of –log(*P*)=7.07 (Fig. 3; Supplementary Figs S2–S4). Gene models containing SNPs above the Bonferroni significance threshold were selected as candidate genes. Significant SNPs for root architecture phenes were located in 15, 19, and 17 unique gene models for water-stress, well-watered, and stress plasticity in Arizona, respectively (Fig. 4; Supplementary Tables S6–S8). Significant SNPs for root architecture phenes were located in 13 and five unique gene models for well-watered conditions in South Africa and environmental plasticity, respectively (Supplementary Tables S9, S10). Of the gene models identified as candidates controlling root phenes in well-watered and water-stress conditions and their plastic responses, ~64% were annotated for MapMan ontogenic categories (Supplementary Tables S6–S10). Environmental plasticity has gene models associated with mainly RNA regulation- and transport-related processes. Stress plasticity has gene models associated with RNA regulation, hormone metabolism, protein degradation, and protein translational modification. Water-stress environments have gene models associated with DNA and RNA regulation, and well-watered environments have gene models associated with abiotic stress and protein degredation in South Africa and protein transport in Arizona (Fig. 5).

A few significant SNPs co-localized between well-watered, water-stress, and/or plastic response groups. Two SNPs co-localized between stress plasticity and water-stress groups. Both of these SNPs are associated with BF and are located in a gene (Zm00001d026191) that is associated with RNA regulation of an ethylene-responsive element-binding protein family (Supplementary Tables S6, S8).

Significant SNPs for well-watered, water-stress, and stress and environmental plasticity groups were detected for all architectural phenes measured, with the exception of DISTLAT. Approximately 46, 22, 24, and 7% of unique gene models were associated with well-watered, water-stress, stress plasticity, and environmental plasticity, respectively (Fig. 4).

A publicly available online resource was developed for users to easily view and explore GWAS results. The website, which can be accessed at https://rootplasticitygwas.nottingham.ac.uk/, provides information about the phenes measured, interactive Manhattan plots, and information about candidate gene annotations and organ and tissue expression patterns in maize and rice, and in Arabidopsis orthologs. Such collective information could help users to prioritize candidate genes for further experimental validation towards understanding the genetic control underlying phenotypic plasticity.

## Discussion

Root architectural phenes are plastic in response to drought and environment. We observed heritable responses of root phenes (Table 2) and large variation in the extent and direction of phene plasticity in response to drought and environment (Fig. 1). Most genetic loci associated with stress or environmental plasticity were distinct from loci controlling phenotypic expression in water-stressed or well-watered conditions (Figs 3, 4; Supplementary Figs S2–S4; Supplementary Tables S6–S10). The genetic architecture and phenotypic characterization of the plastic response of root phenes have important implications in understanding plant adaptation to edaphic stress.

Although significant plasticity was observed in both directions (Fig. 1B), there was no significant overall trend in the direction of plasticity for root angle (ANGLE) between well-watered and water-stress conditions. However, significant ranges of stress and environmental plasticity were observed for root angle (Fig. 1B). In maize, root angle can be plastic in response to nitrogen availability, and in the majority of genotypes studied angles became steeper in nitrogen stress (Trachsel *et al.*, 2013). Steeper root angles enable greater root biomass in deep soil domains and thus greater N capture in leaching environments (Trachsel *et al.*, 2013; Dathe *et al.*, 2016) and increased water capture in drought environments (Mace *et al.*, 2012; Uga *et al.*, 2013). In addition, genetic loci associated with nodal root angle in sorghum co-localized with genes associated with the stay-green drought tolerance mechanism (Mace *et al.*, 2012). DRO1, a gene associated with steep root angle in rice, contributes to avoiding drought stress by increasing deep rooting and thus increasing yield in drought environments (Uga *et al.*, 2013). In wheat, steeper seminal roots were associated with plants with increased drought tolerance (Manschadi *et al.*, 2008; Maccaferri *et al.*, 2016).

Lateral root length (LL) became longer under water stress, and no trend was observed for lateral branching frequency (BF) under stress. Few, long lateral roots compared with many, short lateral roots are beneficial for maize under water and nitrogen stress (Zhan and Lynch, 2015; Zhan *et al.*, 2015). For mobile soil resources, such as nitrogen and water, resource depletion zones surrounding roots are relatively large. Short, dense lateral roots create overlapping resource depletion zones around roots of the same plant, decreasing resource capture efficiency (Ge *et al.*, 2000). Long, dispersed lateral phenotypes along the axial roots optimize the capture of mobile resources as they reduce inter- and intraplant competition (Zhu *et al.*, 2005*c*; Postma *et al.*, 2014; Zhan and Lynch, 2015; Zhan *et al.*, 2015). No significant differences in the mean and range in expression of BF or distance to the first lateral root (DISTLAT) were observed between well-watered and water-stress conditions. While there was no significant overall trend in the direction of plasticity of BF and DISTLAT, a wide range of phenotypic plasticity was observed, and a few individuals had extreme responses to water deficit. Presumably, reduced BF and greater DISTLAT would be beneficial for the capture of mobile resources as they would decrease root length in shallow soil domains, thereby enabling exploration of deeper soil strata (York and Lynch, 2015).

Our results indicate that root architectural phenes and their plastic response to water stress and environment are genetically controlled and highly quantitative. A total of 69 unique gene models were identified as being associated with root architecture within well-watered and water-stressed environments and for stress and environmental plasticity. Many genes with relatively small effect sizes were associated with these phenes, but additively these genes accounted for ~36% of total phenotypic variation. Heritability for phenotypic plasticity was lower than heritability for root phenes in water-stressed and well-watered conditions. This could result from short-term variation in phene plasticity to track fluctuating environmental signals. Heritable plasticity responses indicate that root plasticity is genetically controlled. Heritable plastic responses have also been reported for other species and phenes including flower formation (Bradshaw, 2006; Lande, 2009). Root phenes are highly quantitative, and plasticity in response to edaphic stress and different environments may enable breeding efforts for plastic or non-plastic lines in specific phenes. Understanding root phenotypic plasticity and its genetic control may permit the selection of lines with optimal plasticity to improve plant growth in specific environments. For example, plants with greater phenotypic plasticity for BF may be more useful in environments with fluctuating drought, but reduced phenotypic plasticity for ANGLE may be more beneficial in environments with sustained stress including low nitrogen.

Heritability for root architectural phenes ranged from 0.13 to 0.68 (Table 2). The relatively low percentage of variation explained by significant SNPs (Table 2) can partially be explained by the relatively low heritability of root phenes. However, heritability of field-grown root phenes of mature plants is generally low (Bucksch *et al.*, 2014; Burridge *et al.*, 2016). Low heritabilities may reduce the power of SNP detection, inflate gene effect sizes, and increase the chance of detecting false positives. In the current study, the relatively low heritability values of root phenes can be attributed to the highly heterogenous environment of field-grown maize and the highly quantitative nature of these phenes.

While the overall trend was a decrease in vegetative biomass and yield in drought, significant plasticity was observed in both directions (Supplementary Fig. S5). Plasticity is not simply a growth reduction due to edaphic stress. Plants may have different strategies to achieve drought tolerance, and the plastic responses of root phenes or phene aggregates to drought or different environments could be adaptive or maladaptive. Unresponsiveness of lateral root branching to localized availability of resources, such as water, would be advantageous under drought. Localized proliferation in response to mobile resources, such as water or nitrogen, may be counterproductive as these resources are subject to leaching, movement, and depletion, while root growth is relatively slow and involves significant construction and maintenance costs (Lynch, 2018). In addition, root production in shallow soil domains in response to ephemeral resources may divert resources from root construction in deeper soil domains with greater water availability (Lynch, 2013, 2018).

Specific root phenes are important in plant stress tolerance; however, root phenes do not function in isolation (York *et al.*, 2013; Miguel *et al.*, 2015). Synergisms exist between phene states with a large metabolic cost, for example lateral root branching density, with phenes that reduce the metabolic cost of the root, including fewer basal root whorls in bean. A decreased number of basal root whorls is more beneficial in plants with more dense lateral branching density (Rangarajan *et al.*, 2018). Synergisms exist with phenes that affect the placement of roots in the soil domains. For example, basal root growth angle interacts with root hair density and length to determine the placement of root hairs in the soil profile and increase plant growth up to twice the expected additive effects (York *et al.*, 2013; Miguel *et al.*, 2015). Understanding phene synergisms and their plastic interactions may be an important consideration for breeders.

Auxin has been well studied for its role in regulating root gravitropism (Su *et al.*, 2017) and plays a role in the establishment of root angle (Toal *et al.*, 2018). We identified two candidate genes annotated to auxin-related processes, associated with the phenotypic expression of ANGLE under well-watered conditions (Zm00001d019311; IAA-amino acid hydrolase ILR1-like 7) and stress plasticity (Zm00001d029356; *O*-methyltransferase ZRP4) in Arizona. These genes are orthologs of Arabidopsis *ILR1* (IAA-LEUCINE RESISTANT 1) and ASMT (N-ACETYLSEROTONIN *O*-METHYLTRANSFERASE), respectively. ILR1-like family hydrolases are known to modulate auxin response by regulating auxin homeostasis (Sanchez Carranza *et al.*, 2016). ASMT is involved in the melatonin biosynthetic process, and has been recently implicated in regulating root architecture and gravitropism by modulating auxin response in rice (Liang *et al.*, 2017). Melatonin stimulates several physiological responses to environmental conditions including water deficit (Ding *et al.*, 2018; Debnath *et al.*, 2019).

Another candidate gene associated with the phenotypic expression of DISTLAT under stress plasticity (Zm00001d024644) is a MYB-related transcription factor family protein. Due to its role in auxin biosynthesis, the overexpression of this MYB gene showed a significantly increased number of lateral roots and elongated hypocotyl (Kwon *et al.*, 2013). Auxin has known roles in the establishment of root angle and development of lateral roots, and presumably is an important regulator of the development of other root phenes.

A candidate gene associated with lateral root branching frequency (BF) under well-watered conditions in South Africa (Zm00008a029231) is a GRAS transcription factor family protein. Its Arabidopsis ortholog encodes SHR (Short Root), is involved in asymmetric cell division and radial pattern formation in root, and is required for the initiation and patterning of lateral root primordia (Lucas *et al.*, 2011). Additionally, our study associated Zm00001d043612 (orthologous to Arabidopsis *FLZ10*) with BF under well-watered conditions in Arizona. This gene is expressed in young primordia during lateral root development and its mutant showed reduced lateral roots (Jamsheer *et al.*, 2018). The expression of *FLZ* genes is highly regulated by energy status and abiotic stress (Jamsheer and Laxmi, 2015).

Cytokinin metabolism and signaling genes are known to form a redundant network to modulate lateral root initiation and outgrowth of young primordia. A candidate gene associated with the stress plasticity response of LL is involved in cytokinin metabolism and signaling. Cytokinin is also known to act antagonistically to other hormones (e.g. brassinosteroids) and affect lateral root elongation in cytokinin receptor mutants (Chang *et al.*, 2015). Multilevel redundancy of cytokinin modulating lateral root phenes is believed to reflect the role of cytokinin in mediating environmental signals.

A candidate gene associated with the phenotypic expression of DISTLAT under stress plasticity (Zm00001d038366) is involved in lipid metabolism. The Arabidopsis ortholog of this gene encodes GPAT5, a glycerol-3-phosphate SN-2-acyltransferase that is involved in suberin biosynthesis. GPAT5 was found to be specifically expressed in the lateral root formation zone in the root and may be required for lateral root formation (Beisson *et al.*, 2007). Suberin functions as an apoplastic diffusion barrier at lateral root emergence sites (Li *et al.*, 2017), and developmental variations of apoplastic barriers within lateral root system could be an important trait in response to abiotic stress factors (Tylová *et al.*, 2017).

A cytochrome P450 gene differentially expressed in nitrogen stress conditions in maize leaves and ears (Zm00001d048702) (Arp, 2017) has implications in auxin formation (Irmisch *et al.*, 2015) and was associated with DISTLAT in well-watered conditions. Lateral branching phenes including LL and BF have roles in plant performance under nitrogen stress (Zhan and Lynch, 2015). Presumably, DISTLAT also influences plant performance in edaphic stress by affecting the metabolic cost of soil exploration (York *et al.*, 2015). A gene controlling aquaporin expression in maize (Zm00008a000537) (Yue *et al.*, 2012) was associated with the plasticity of LL between different environments. LL has been demonstrated to have a large role in drought tolerance (Zhan *et al.*, 2015) and presumably is associated with genes controlling drought tolerance including aquaporin genes. A gene associated with environmental plasticity of root angle (Zm00008a014805) was up-regulated in leaves in water deficit and cold stress (F. Liu *et al.*, 2016). Root angle influences rooting depth and thus the capture of deep soil water, increasing drought tolerance (Mace *et al.*, 2012; Uga *et al.*, 2013).

A gene associated with the plasticity of root angle in different environments (Zm00008a038792) was up-regulated during phosphate starvation in maize (Xu *et al.*, 2018). A gene associated with stress plasticity of DISTLAT (Zm00001d038366) is up-regulated during phosphorus stress and is involved in phosphorus metabolism and utilization (Yu *et al.*, 2019), and may also be involved in suberin synthesis (Zhu *et al.*, 2019). A number of root phenes influence phosphorus capture under suboptimal phosphorus availability, including the density of lateral branching (Zhu and Lynch, 2004; Postma *et al.*, 2014), root cortical aerenchyma (RCA) (Galindo-Castañeda *et al.*, 2018), root angle (Zhu *et al.*, 2005*a*), the number of crown roots (Sun *et al.*, 2018), and root hair length (Zhu *et al.*, 2010). Presumably, genes that are differentially expressed in low phosphorus availability could contribute to controlling root phenes under many edaphic stresses through common signaling pathways such as auxin and ethylene (Ma *et al.*, 2003; Giri *et al.*, 2018).

A gene associated with the plastic response of BF and the phenotypic expression of BF in water stress (Zm00001d026191) co-localized with a gene up-regulated in maize cortical cells upon ethylene exposure which encoded an ethylene response factor class of transcription factor (Takahashi *et al.*, 2015). A gene associated with BF in well-watered conditions is annotated to APETALA2/Ethylene-responsive element binding protein (AP2/EREBP). A differentially expressed gene in root cortical cells during ethylene-induced RCA formation (Zm00008a029231) (Takahashi *et al.*, 2015) was associated with BF in well-watered conditions in the South Africa field site. Hypoxia induces RCA formation (Evans, 2003), and presumably common signaling pathways (e.g. ethylene) induce RCA formation and control expression of other root phenes under a range of edaphic stresses. For example, ethylene inhibits root branching at the earliest stages of lateral root initiation (Negi *et al.*, 2008; Lewis *et al.*, 2011).

Of the gene models identified, 45% were annotated. The stress plasticity group had significantly more genes associated with hormones and abiotic stress compared with the well-watered and water-stressed groups (Fig. 5). Hormone signaling, particularly ABA and ethylene signaling, has important implications in drought tolerance (Sharp and LeNoble, 2002; Shi *et al.*, 2015). In addition, a gene associated with cytokinin signal transduction (Zm00001d037694) was associated with the water stress response of lateral root length. Cytokinin signal transduction is associated with adaptation to stress and interacts with ABA signaling (Ha *et al.*, 2012). The co-localization of a few SNPs between the well-watered, water-stressed, and stress and environmental plasticity groups indicates that plasticity is controlled by many different genes in distinct pathways (Figs 4, 5).

The fact that there was no co-localization of significant SNPs between the Arizona and South Africa field sites for the same root phenes indicates that the expression of these phenes, and subsequent identification of associated genetic loci, is highly dependent on the environment. Factors including differences in soil texture, photoperiod, and irrigation regimes may account for some of these differences. In the South Africa environment, root angles were steeper and lateral root phenes had considerably less variation compared with the Arizona environment. Highly quantitative traits with small effects, such as root architectural phenes, may not be ideal for GWAS models that have historically been successful with qualitative traits or highly heritable quantitative traits (e.g. flowering time). Consideration of multiple phenes, gene networks, and dynamic responses may result in stronger associations of phenes with genetic loci or regulatory pathways.

Environmental plasticity has widespread implications in interpretation and extrapolation of data from different growing systems and environments. With a few notable exceptions (e.g. Schneider *et al.*, 2020), the majority of root quantitative trait loci (QTLs) and GWAS studies use artificial growth systems (Zhu *et al.*, 2005*c*, 2006; Trachsel *et al.*, 2009) that do not realistically represent field conditions and therefore cannot adequately address questions related to root architecture and its relationship to nutrient uptake. In addition, many studies examine embryonic root systems (Hund *et al.*, 2004; Zhu *et al.*, 2005*c*; Trachsel *et al.*, 2009), which are poor predictors of mature root system architecture (Zhu *et al.*, 2011) and may be under genetic control distinct from that of post-embryonic root systems (Hochholdinger and Feix, 1998; Hochholdinger *et al.*, 2001). Root growth in artificial systems may be constrained by the size of the growth media or container, and is buffered from the atmospheric environment in a completely different way when compared with field-grown conditions. In addition, elongation and trajectory of growing roots are affected by changes in soil bulk density as a result of sieving and compacting soil, relative to undisturbed soil. The spatiotemporal dynamics of nutrient and water regimes in soil are difficult to mimic in artificial media. Therefore, root growth in artificial media may be artifactual relative to field conditions (Walter *et al.*, 2009).

In this study, root architectural phenotypes were profiled in diverse maize lines with and without water stress in the field in multiple environments. Significant and substantial variation was observed for all phenes in well-watered and water-stressed environments, and for plasticity, namely phenotypic responses to water availability or phenotypic responses to different environmental conditions. Root architectural phenes and their responses to drought and environment are heritable and genetically controlled. Phenotypic plasticity and interactions between root phenes may be synergistic or antagonistic. Phenotypic plasticity and interactions between phenes will require further research to understand their implications for edaphic stress tolerance. Identifying genes that control root phenes and their plastic expression under edaphic stress will enable the selection of lines with greater or reduced plasticity in breeding programs to increase plant productivity. Identification of genes underlying root plasticity can provide breeders with novel opportunities to develop crop varieties better suited to a wide range of environments and agroecosystems.

## Supplementary data

Supplementary data are available at *JXB* online.

Table S1. Genotypes in the Wisconsin Diversity Panel grown in Arizona.

Table S2. Genotypes in the Wisconsin Diversity Panel grown in South Africa.

Table S3. Phenotypic variation of root architectural traits.

Table S4. Summary of the analysis of variance for root architectural phenes.

Table S5. Allometric analysis of root phenes.

Table S6. Gene models identified for root phenes in water-stressed conditions in Arizona.

Table S7. Gene models identified for root phenes in well-watered conditions in Arizona.

Table S8. Gene models identified to control plasticity in drought for root phenes.

Table S9. Gene models identified for root phenes in well-watered conditions in South Africa.

Table S10. Gene models identified to control environmental plasticity for root phenes.

Fig. S1. Q-Q plots assessing the fitness of K model for GWAS of root phenes.

Fig. S2. GWAS results for DISTLAT for plants grown in well-watered and water-stress conditions, and their plasticity.

Fig. S3. GWAS results for BF for plants grown in well-watered and water-stress conditions, and their plasticity.

Fig. S4. GWAS results for LL for plants grown in well-watered and water-stress conditions, and their plasticity.

Fig. S5. Violin plots showing the distribution of vegetative biomass and yield.

eraa084_suppl_Supplementary_Figure_S1_S5

eraa084_suppl_Supplementary_Tables_S1_S10

## Abbreviations

ANGLE: angle of roots relative to the soil line
BF: lateral branching frequency on the excised root
DISTLAT: distance to the first lateral root from the root apex on the excised root
GWAS: genome-wide association study
LL: average lateral root length
QTL: quantitative trait locus
WS: water stress
WW: well-watered
