# Supplementary material for: Genetic control of root architectural plasticity in maize
Source: J Exp Bot. 2020 Feb 21;71(10):3185–97. doi: 10.1093/jxb/eraa084 (PMC7260711; doi:10.1093/jxb/eraa084)

Supplemental Figure 1. Q-Q plots assessing the fitness of K model for GWAS of root phenes for A) plasticity B) well-watered and C) water-stress.

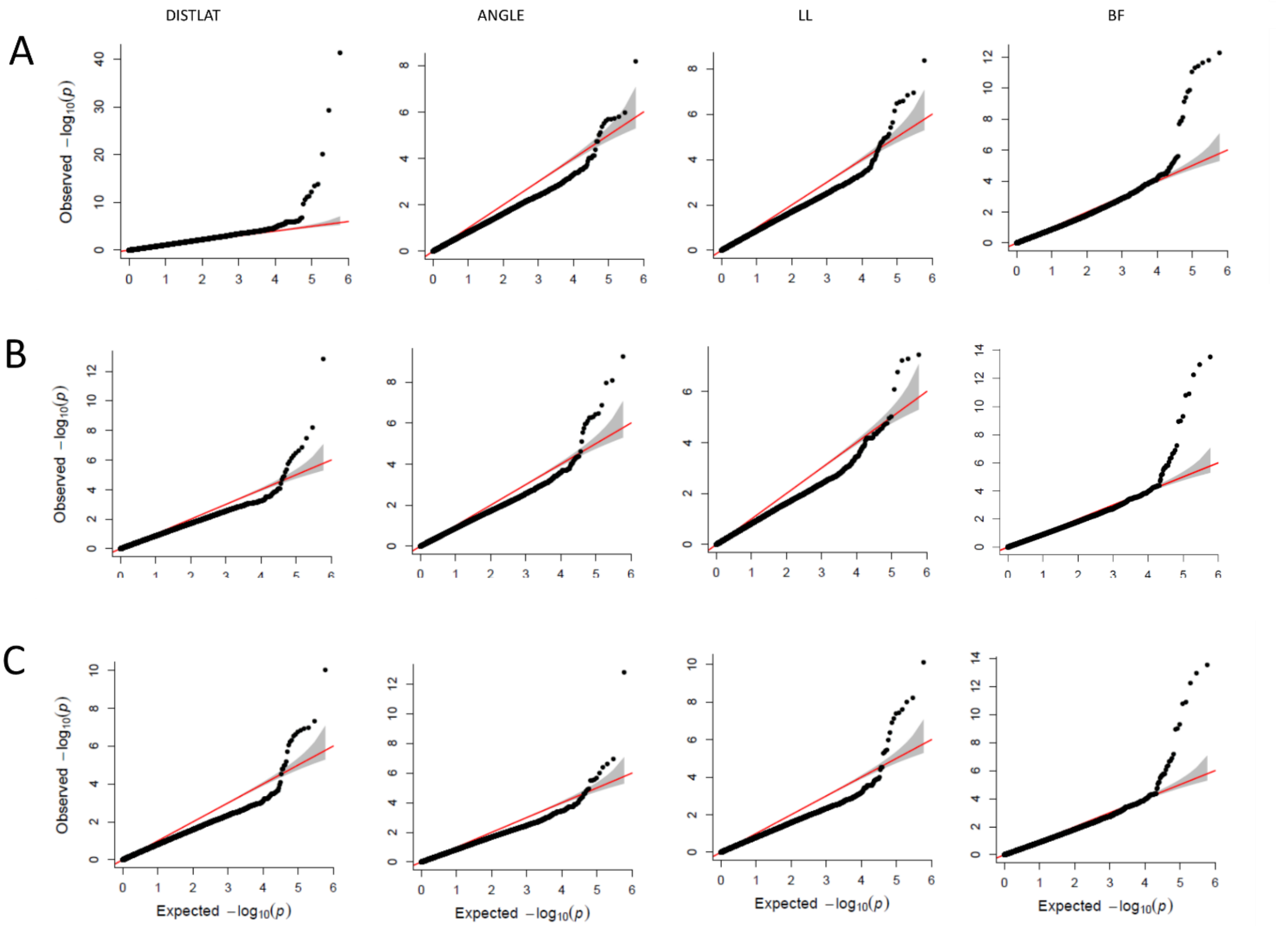

Supplemental Figure 2. GWAS results for root angle (DISTLAT) for plants grown in A) well-watered conditions B) water stress conditions C) water-stress plasticity in Arizona D) well-watered conditions in South Africa E) environmental plasticity. See supplementary figure 2 for plots for other architectural phenes

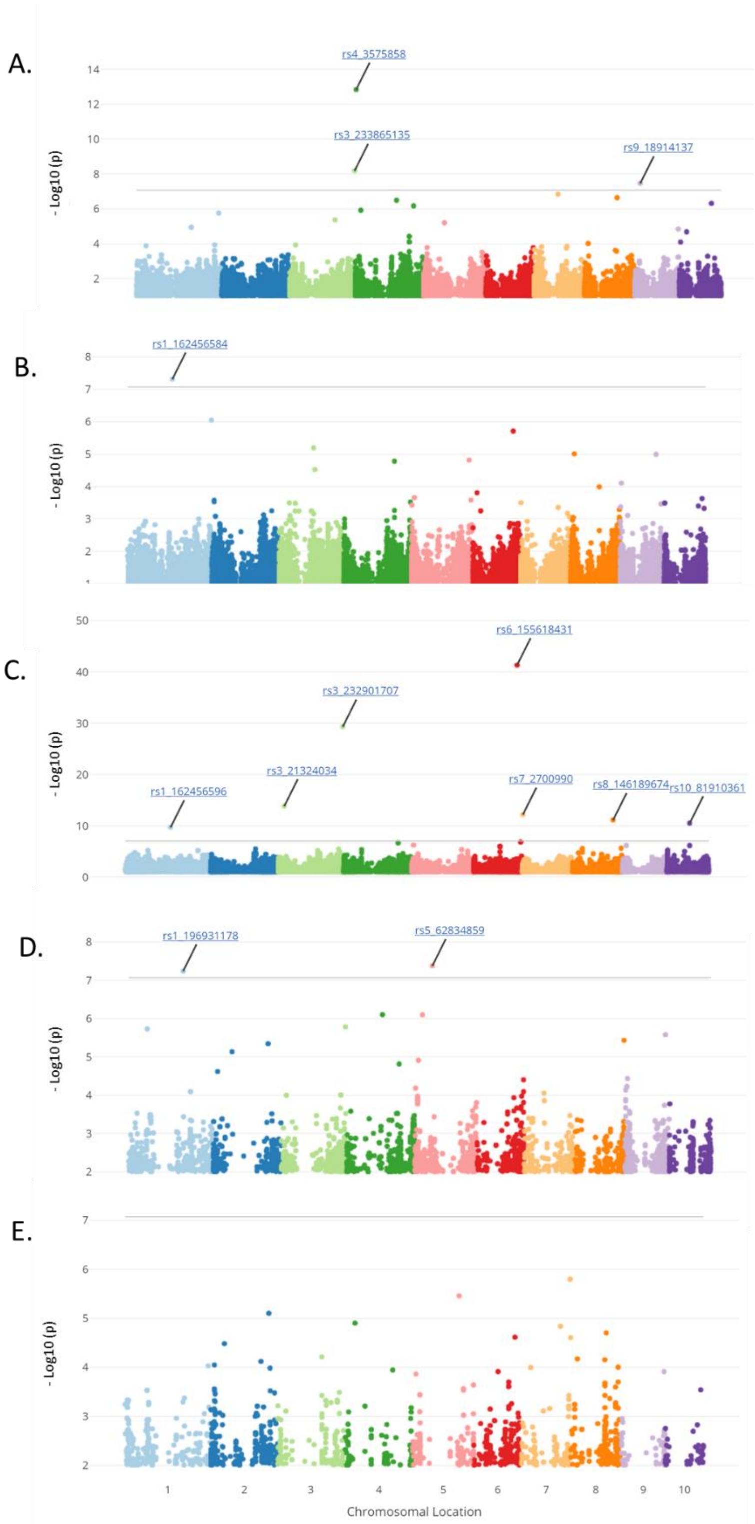

Supplemental Figure 3. GWAS results for root angle (BF) for plants grown in A) well-watered conditions B) water stress conditions C) water-stress plasticity in Arizona D) well-watered conditions in South Africa E) environmental plasticity. See supplementary figure 2 for plots for other architectural phenes

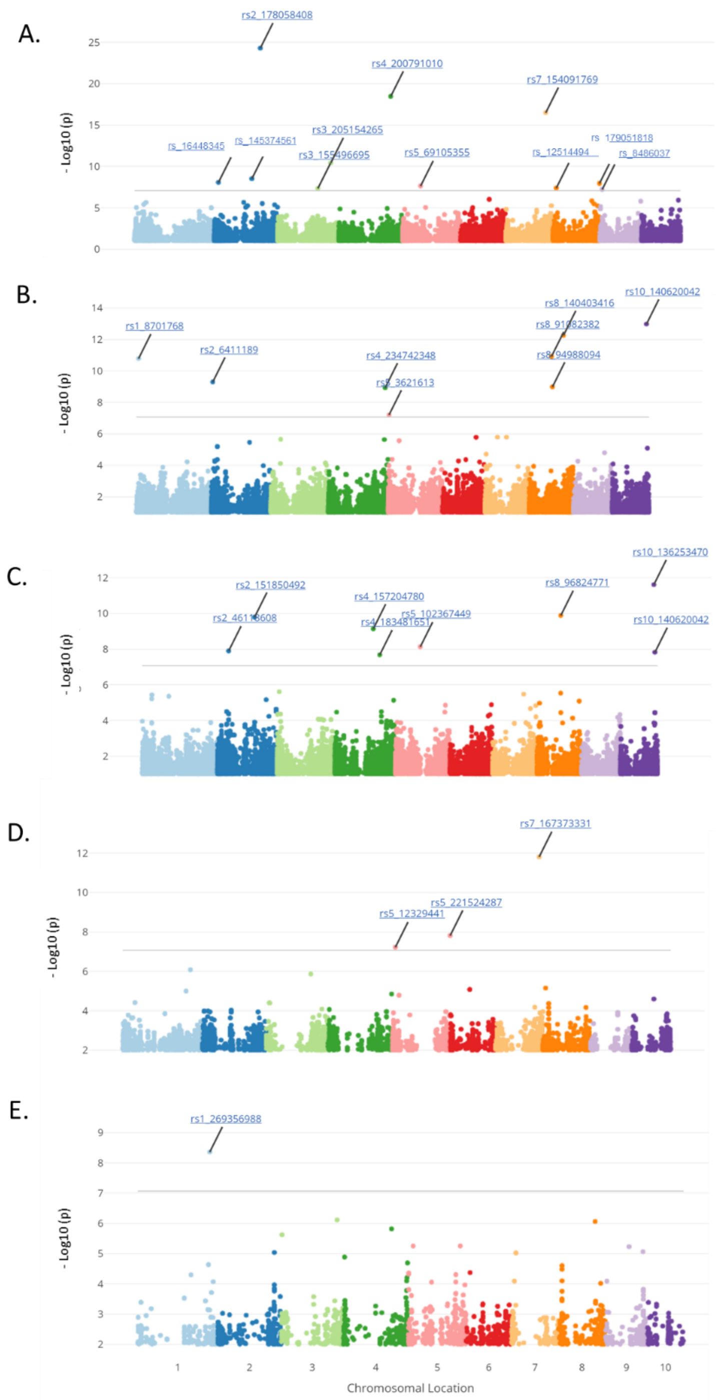

Supplemental Figure 4. GWAS results for root angle (LL) for plants grown in A) well-watered conditions B) water stress conditions C) water-stress plasticity in Arizona D) well-watered conditions in South Africa E) environmental plasticity. See supplementary figure 2 for plots for other architectural phenes

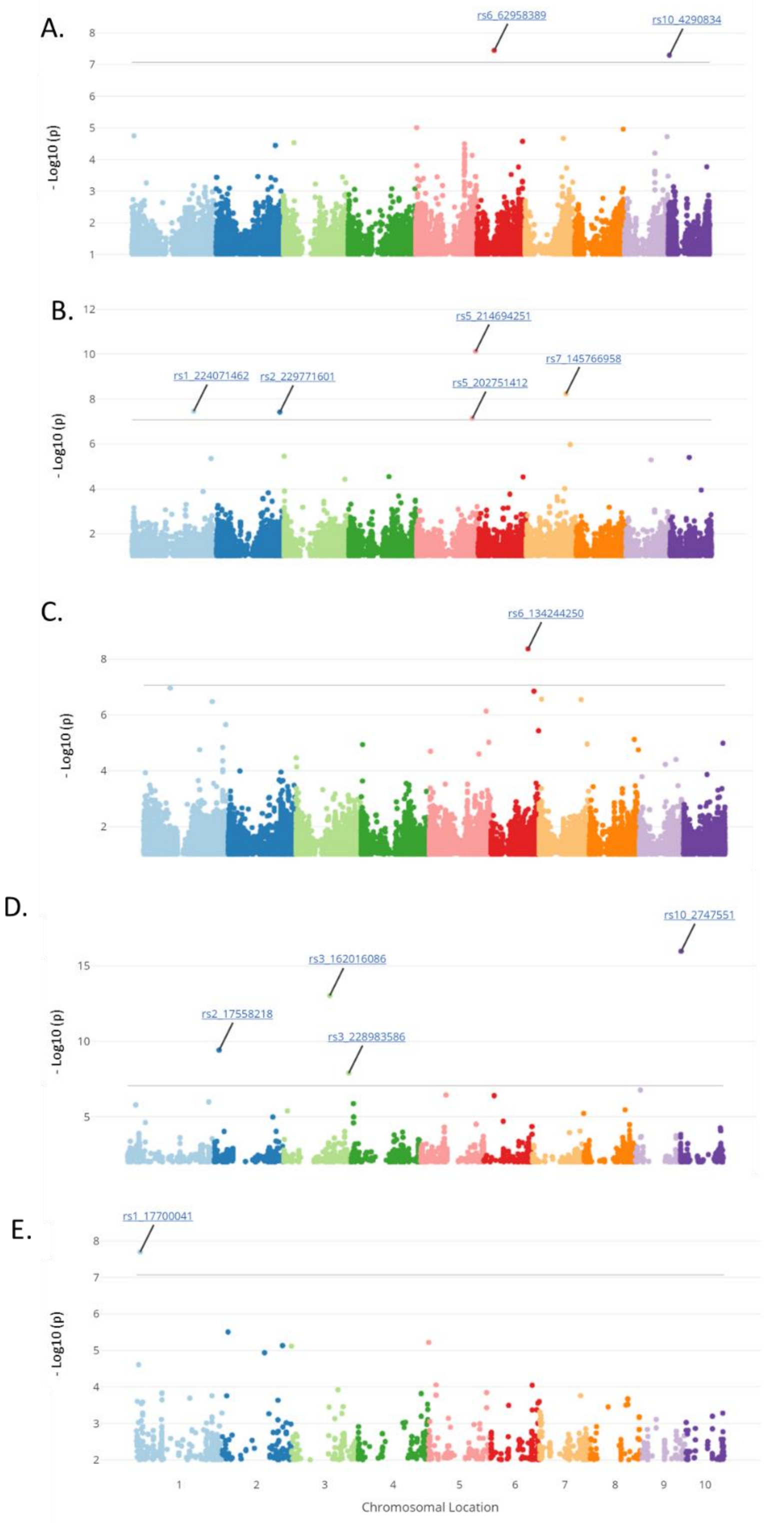

Supplemental Figure 5. Violin plots showing the distribution of vegetative biomass (Bio_Veg) and yield in A) well-watered (blue), water-stressed (red), and B) the plastic response to drought.

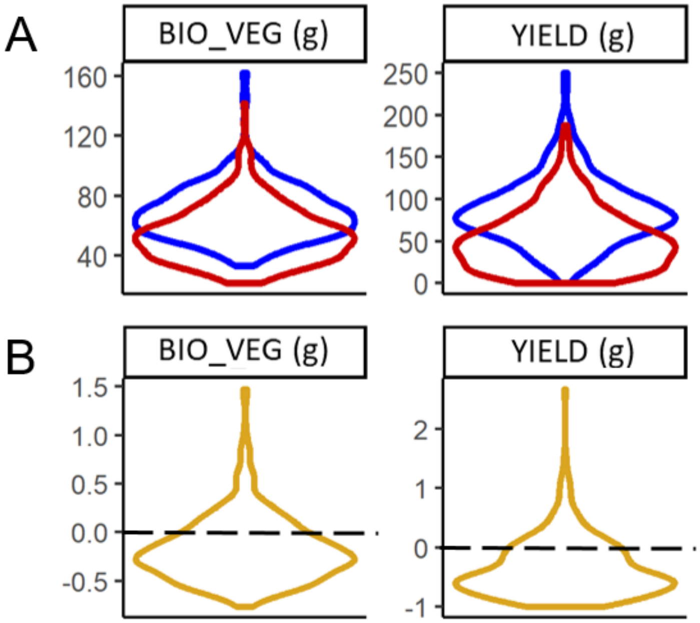

Supplement: eraa084_suppl_Supplementary_Figure_S1_S5 [file eraa084_suppl_supplementary_figure_s1_s5.pdf]
